# Ecological Restoration Strategies for Mountainous Cities Based on Ecological Security Patterns and Circuit Theory: A Case of Central Urban Areas in Chongqing, China

**DOI:** 10.3390/ijerph192416505

**Published:** 2022-12-08

**Authors:** Liang Lv, Shihao Zhang, Jie Zhu, Ziming Wang, Zhe Wang, Guoqing Li, Chen Yang

**Affiliations:** Key Laboratory of New Technology for Construction of Cities in Mountain Areas, School of Architecture and Urban Planning, Chongqing University, Chongqing 400044, China

**Keywords:** ecological networks, ecological restoration, ecological barrier points, ecological “pinch points”, GIS

## Abstract

Urban ecosystems are under enormous pressure in the background of rapid urbanization. Mountainous cities are more prone to degradation due to dramatic topography. Ecological security patterns combined with circuit theory can effectively identify ecological barriers and “pinch points” and propose targeted ecological restoration and protection strategies. In order to ensure the ecosystem health and sustainable development of mountainous cities, this paper applies the MSPA model, Invest model, MCR model, and Linkage Mapper Tools to identify the ecological source regions, eco-corridors, and “key points” in the central metropolitan area of Chongqing. The study shows that: (1) There are 43 ecological sources in the central urban area of Chongqing, with a total area of 986.56 km^2^, and it forms a linear distribution with a multi-patch scattering pattern. (2) A series of 86 ecological corridors in the area, totaling 315.14 km, show a pattern of more corridors in the east and fewer in the west. (3) The research found 17 sites totaling 24.20 km of the ecological corridor in the barrier point zone. In addition, up to 22 segments, totaling 19.27 km of the ecological corridor, are located in the “pinch point” zone. (4) The barrier point and “pinch point” on the ecological corridors are identified to obtain their type, scale, and location, thus suggesting conservation-restoration.

## 1. Introduction

The growth of the urban population and the rapid development of urbanization rates may have negative impacts on the sustainability of urban ecology [1]. As is currently happening in China, cities have entered a phase of accelerated expansion after the reform and opening up, and their urbanization rate has reached 60.6% by 2020 from 17.8% in 1978. The urbanization rate in southeastern coastal areas such as Shenzhen, Foshan, Dongguan, and Zhuhai has even exceeded 80%, the level being on par with that of developed countries [2]. In the process of modernization and population growth, the lack of experience has caused a series of problems, such as tightening resource constraints, severe environmental pollution, and ecosystem degradation. Building an ecological civilization is presented in this context as a national policy, a holistic strategy for the future of human living conditions [3]. As a result, ecological civilization has been raised to an unprecedented level, while the theme of ecological restoration has become the subject of future exploration by researchers [4].

Research on ecological restoration is currently the focus of exploration in the disciplines of ecology and the human environment, involving micro-engineering restoration and macro-ecological assessment. Researchers have concentrated on water and ecological restoration, soil and water conservation, wetland regeneration, and eco-restoration of mining areas at the micro level. For micro-level studies, scholars prefer to restore single ecological issues through the application of engineering techniques, such as ecological restoration of polluted water bodies based on chemical, biological, and engineering methods [5,6,7]; ecological maintenance of soil and water resources through converting cultivated land into forests and reforestation [8,9]; contribution to the cycle of wetland ecosystems by preserving key species [10]; and zoned reclamation approaches have also been used to restore mining areas ecologically [11].

From the macro perspective, experts mainly assess the ecological sensitivity [12], ecological conservation importance [13], degree of ecosystem degradation [14], and ecological vulnerability [15] of the region and on its foundation, make the classification of ecological restoration zoning and ecological restoration levels. Based on the results of the above classification, the priority of ecological restoration can be determined, the essence of which is to enhance the ecological quality of the environmentally damaged area, which in turn has a positive impact on the entire natural landscape of the locality.

In general, the micro approach is mainly based on engineering techniques. It emphasizes the mitigation of a particular ecological risk and lacks consideration of the overall pattern. However, the macro strategy offers more insight into the ecological status but less into the processes. Moreover, it improves the system’s resilience as a group by addressing the poorer environmental situation. Therefore, ecological restoration should follow a holistic to microscopic approach, which allows the connectivity of ecological flows to be fully considered and the ecological goals of low-cost maintenance to be achieved by improving the constraints in critical areas.

Ecological security patterns are based on the ‘source-sink’ theory and have developed an “ecological source identification—building a resistance surface—extracting corridors paradigm” [16]. This approach reflects the basic idea of ecological patterns and integrates the analysis of development processes. Since 1990, when the ecological security landscape was proposed, it has been seen as a framework for maintaining ecological security and sustainable urban development and has been practiced extensively [17]. For example, the World Wide Fund for Nature (WWF) delineated 200 priority protected areas and identified a series of representative ecosystems in the early 20th century; The Conservation International (CI) identified 35 biodiversity conservation hotspots to address threatened habitats; the World Conservation Union (IUCN) identified vital biodiversity areas. Current research on ecological security patterns focuses on constructing ecological security patterns and optimizing of ecosystems [18]. In the construction of ecological security patterns, the extraction of ecological sources and the identification of ecological corridors are the primary research. Early researchers tend to directly select large patches and nature reserves as ecological sources [19,20]. However, it has developed to identify ecological sources by quantitative methods such as Invest model (eco-environmental quality) [21], morphological spatial pattern analysis (MSPA) [22,23], or constructing evaluation systems [24,25]. Recently, more scholars have shown that combining two or more methods can effectively extract ecological sources. For example, Sucui Li identified ecological sources in the eastern part of Ordos City, Inner Mongolia Autonomous Region, China, by combining the Invest model and MSPA [24]; Feng Tang et al. used the same method to identify the ecological security pattern in the Huaiyang section of the Grand Canal [26]. For corridor extraction, the MCR(minimum cumulative resistance) has been widely used for ecological flow simulation based on landscape heterogeneity [27]. Ecological corridors are identified by MCR models based on the construction of ecological resistance surfaces. Researchers have mainly used land use as the resistance surface. However, due to the high fragmentation of urban landscapes, the diversity of land use patterns, and the complexity of the interactions between land use and ecological processes, homogeneous assignments based on land cover types inevitably mask spatial heterogeneity among the same areas. As a result, land use does not accurately reflect ecological resistance. In recent years, scholars have tried introducing spatial data such as nighttime light data, vegetation cover, or slope to correct the ecological resistance coefficient among single land cover types [28,29]. In terms of ecological optimization, there are apparent differences in proposed optimization strategies because of scholars’ different claims. For example, Yang Junfu optimized the ecological security pattern of the Loess Plateau in China through landscape index [18], Shi H et al. optimized the ecological security pattern of Tianchi in Xinjiang based on landscape pattern [30], and Liang Fachao et al. optimized the ecological security pattern of Quanzhou, China from the perspective of ecological environment quality [31]. In general, the current optimization strategies based on ecological security pattern focus on optimizing ecological security patterns through landscape index, pattern, and ecological environment quality. These studies have important implications for sustainable urban development, but these opinions are often too macroscopic to determine the location, type, and amount of specific restoration. Thus, they cannot be applied well to urban ecological restoration practices.

Furthermore, with the emergence of circuit theory and its effectiveness in identifying negative factors [21,32], the collaborative work of ecological security patterns and this theory provides practical possibilities for restoring ecological key points and the positive circulation of ecological flows. Circuit theory has its roots in physics and has been applied to studying of gene flow in heterogeneous landscapes. Brad McRae’s team of senior landscape ecologists at The Nature Conservancy developed Linkage Mapper software to predict movement patterns in complex landscapes and to accurately identify the location, number, and type of ecological barriers and “pinch points” [33,34,35]. It could achieve large-scale ecological restoration outcomes through less costly inputs.

Mountains cover a quarter of the Earth’s surface, and nearly 12% of the world’s population lives in mountainous and high-altitude areas [36]. Globally, the population living in mountainous urban areas is gradually increasing). For example, in developing countries, more than a quarter of the mountain population lives in cities [37]. The ecological attributes of the mountainous cities are enormously different from those of the plains [38,39]. The dramatic topography of mountainous cities leads to their ecological fragility and biodiversity characteristics. It is susceptible to more significant damage from human disturbance and has high ecological rehabilitation costs. The destruction of some sources will negatively impact the overall ecological environment because of the more systematic character of the ecological sources in cities made up of continuous mountains and water systems [40,41]. In summary, restoring ecological key points to improve the integrity of a complex mountainous urban ecology is well suited for this purpose. However, a search of WOS under the titles of “mountainous cities” and “Ecological restoration” showed that there were few studies on ecological restoration of ecological security patterns in mountainous cities.

Therefore, the focus of this study is to combine the construction of ecological security patterns with circuit theory to identify ecological key points in Chongqing, a mountainous city, and to determine their location, scale, and type. The whole project will be taken forward to propose targeted ecological restoration strategies, and its application may provide theoretical support and practical reference for future ecological restoration of complicated terrain.

## 2. Materials and Methods

### 2.1. Study Area

**Figure 1:**
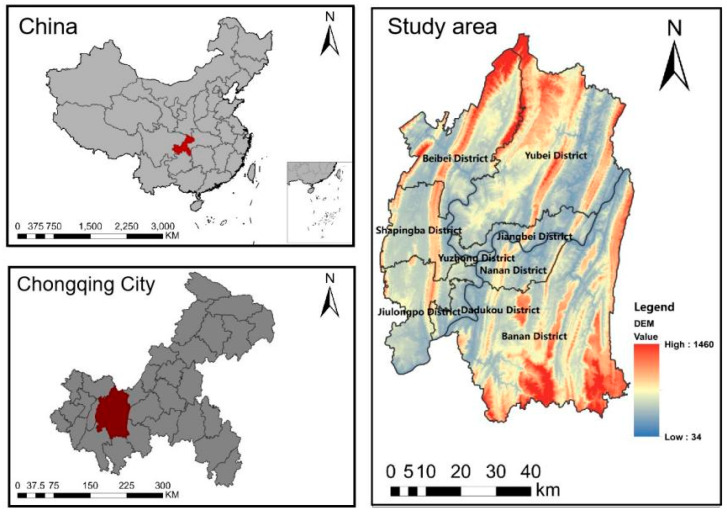
Situation of the study cases.

The study area (Figure 1) belongs to the central region of Chongqing (29°8′2″ N–30°7′37″ N, 106°14′49″ E–106°58′26″ E) and contains a total of nine administrative districts, namely Yuzhong, Yubei, Shapingba, Nanan, Jiulongpo, Jiangbei, Dadukou, Beibei and Banan districts. It is about 34–1460 m above sea level and has a population of 7,178,000, which is 20.9% of the city’s total population. Moreover, it covers an area of about 5400 km^2^ accounting for about 6.6% of the city’s area [42]. The unique topographical characteristics of the study area have created an exceptional ecological environment, and this typical mountainous city is an invaluable object of study for scholars. In the last ten years, the population in this area has increased by 16.4%, and the GDP has risen by 203.82% [42,43]. The rapid population and economic growth have directly or implicitly led to issues such as soil erosion, urban heat island, and green space fragmentation in the region [44].

### 2.2. Data Source

As shown in Table 1, the study data contains five main components. (1) Land use data covers ten land use types: cultivated land, woodland, grassland, shrubland, wetland, water body, tundra, artificial ground, bare ground, and glacier, mainly used in Morphological Spatial Pattern Analysis, eco-environmental quality evaluation and resistance surface analysis. (2) GDEMV3 30M resolution digital elevation data is collected as a source for obtaining slope and topographic relief factors. (3) The vegetation cover of the study area is obtained from Landsat 8 OLI_TIRS satellite digital products. (4) The road network data is based on the Open Street Map platform, which contains mainly vector data for roads and railways. (5) The administrative boundary data is acquired through vectorization of the Territorial Spatial Planning of Chongqing Municipality (2019–2035). Ultimately, the five primary data types are processed according to the different requirements of each section, and the relevant calculations are performed with raster data of 30 × 30 accuracy.

### 2.3. Principles of Ecological Restoration Based on Ecological Security Patterns and Circuit Theory

**Figure 2:**
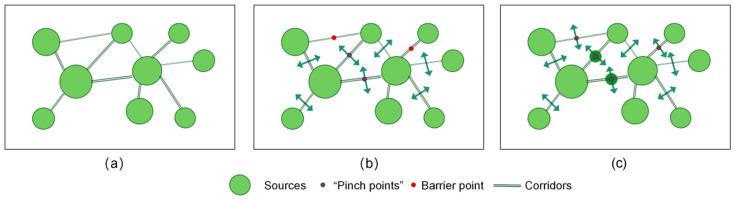
Principles of ecological network optimization. (**a**) Construction of ecological security pattern. (**b**) Ecological key point identification. (**c**) Optimizing Ecological Network.

The network constructed by key sources and corridors functions continuously by undertaking biological migrations, which are essential for regional ecological security and stability (Figure 2a). Concerning ecological corridors, two key points significantly influence the operation of ecological functions. One of these is the barrier point, which prevents the normal circulation of natural flows and requires removal for the broader ecosystem to develop a positive cycle. Furthermore, ecological “pinch points” are vital nodes in ecological corridors, the disruption of which affects the resilience of the whole system (Figure 2b). Hence, a comprehensive consideration of ecological restoration theory based on key points can optimize the entire ecological network and enhance the overall environmental quality by removing barriers and protecting “pinch points” along the ecological corridor (Figure 2c).

### 2.4. Research Framework

**Figure 3:**
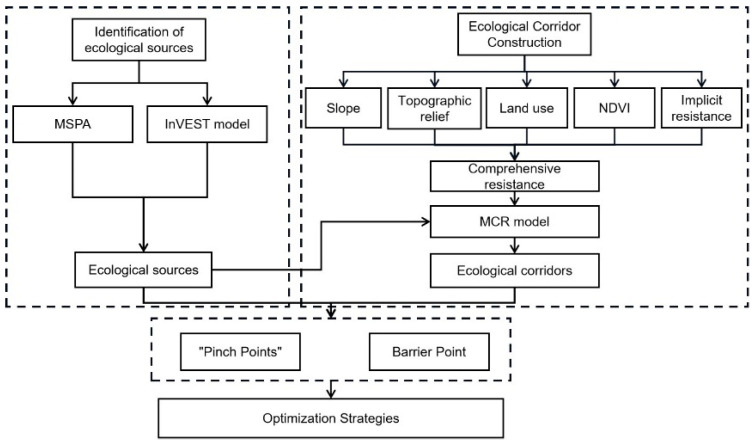
Framework of this study.

As shown in Figure 3, the research framework is divided into the following three parts: (1) Identification of ecological sources: considering the environmental quality and morphological characteristics, the ecological core areas extracted by MSPA are combined with the high environmental quality areas selected by the InVEST model to identify the ecological sources. (2) Spatial analysis of ecological corridors: Combining multiple factors to calculate the comprehensive resistance surface. After that, the ecological corridor is calculated by MCR model. (3) Linkage Mapper Tools identifies key points in the ecological corridor and suggests optimization strategies based on location, scale, and type.

### 2.5. Methodology

#### 2.5.1. Identification of Ecological Sources

1. Evaluation of Ecological Quality

The US Natural Capital Project Team developed the InVEST model, and its Habitat Quality module is adequate for ecosystem evaluation and habitat quality analysis. In the evaluation process, the InVEST model can synthesize the relative sensitivity of habitat types to each threat factor and their relative impacts, facilitating the quantitative analysis of habitat [45]. Its calculation formula is as follows [46]:

(1) $$Q_{\mathrm{xj}\;={\;H}_{j}}\left(1-\left(\frac{D_{\mathrm{xj}}^{z}}{D_{\mathrm{xj}}^{z}+K^{z}}\right)\right)$$

where $Q_{\mathrm{xj}}$ represents the habitat quality index of raster x in habitat type j, with $Q_{\mathrm{xj}}$ values ranging from 0 to 1; $H_{j}$ is the habitat suitability weights of habitat type j; $D_{\mathrm{xj}}^{z}$ indicates the habitat degradation of raster x in habitat type j; z refers to the scale constant, with a default value of 2.5; and K as the half-saturation constant is half of the highest habitat degradation raster value.

After referring to previous studies on ecological environment quality [47,48], the ecological threat sources and impact categories are determined in the context of the actual situation in the central metropolitan area of Chongqing (Table 2). Meanwhile, the ecological sensitivity of habitat types to different threat sources is set (Table 3), and the eco-environmental quality of Chongqing is calculated on this foundation through the Habitat Quality module in InVEST 3.8.0.

2. Morphological Spatial Pattern Analysis

MSPA is a mathematical morphology-based classification process proposed by Vogt et al. It classifies binary raster images into seven elements: Core, Islet, Perforation, Edge, Bridge, Loop, and Branch, based on the principles of erosion, geodesic dilation, and Euclidean distance thresholds between raster cells (Table 4) [49,50,51]. In this operation, woodlands, watersheds, wetlands, and grasslands with positive ecological services are treated as “foreground,” and the rest of the areas are selected as “background” for analysis. The 30 × 30 m raster map of land use categories is converted into a binary image of foreground and background using the ArcGIS platform and then processed into seven types of landscape elements using the Guidos Toolbox software (Peter Vogt, European Commission, Joint Research Centre (JRC), Ispra (VA), Italy).

#### 2.5.2. Spatial Analysis of Ecological Corridors

Ecological corridors are low-resistance pathways between ecological sources, promoting the migration of biological flows between sources. The MCR model proposed by Knaapen et al. is currently applied in a wide range of fields, such as urban planning and constructing ecological security patterns, due to its practicality and scalability [52,53,54]. The model identifies the lowest resistance path between the two sources as a potential ecological corridor by calculating the resistance that needs to be overcome to get from the source to the destination with the following formula.

(2) $$\mathrm{MCR}=f_{\min}\overset{1=m}{\underset{f=n}{\sum }}D_{\mathrm{ij}}\times R_{i}$$

where MCR is the minimum cumulative resistance value $D_{\mathrm{ij}}$, which represents the spatial distance of species from the ecological source to landscape unit i; $R_{i}$ indicates the landscape unit’s resistance coefficient to the species’ migration. f denotes the positive correlation between minimum cumulative resistance and ecological processes. Traditional resistance surfaces are constructed by simulating ecological resistance based on the land use characteristics of patches. In order to assess resistance more accurately, this paper draws on relevant studies [33,52,53,55,56] to incorporate parameters such as implicit ecological resistance values, land use, slope, and topographic relief, NDVI and obtain weights through the AHP method (Table 5).

#### 2.5.3. Identification of Ecological Key Points

The Linkage Mapper Tools, developed by Brad McRae’s team of senior landscape ecologists at The Nature Conservancy, has been used extensively in ecological conservation planning and combines various analytical tools. As shown in Table 6, Barrier Mapper, and Pinchpoint Mapper are chosen to simulate the transfer pathways and their key points in this study [33,34,35].

## 3. Results

### 3.1. Identification of Ecological Sources

**Figure 4:**
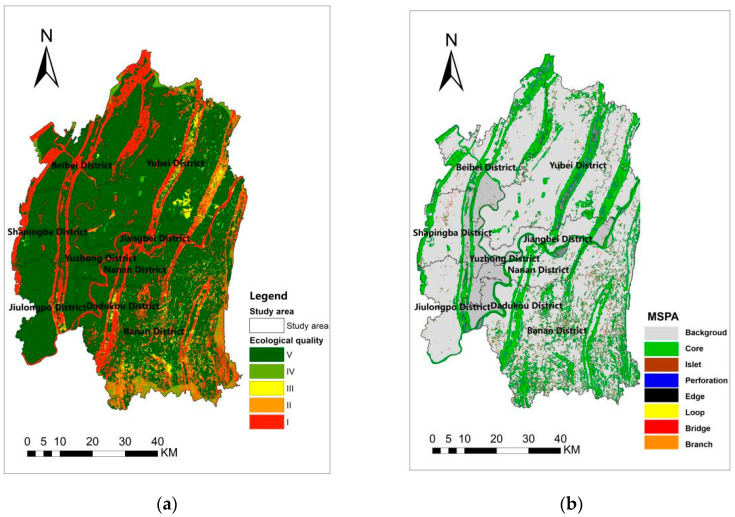
Ecological quality distribution and MSPA landscape pattern (**a**) Ecological quality distribution (**b**) MSPA landscape pattern.

**Figure 5:**
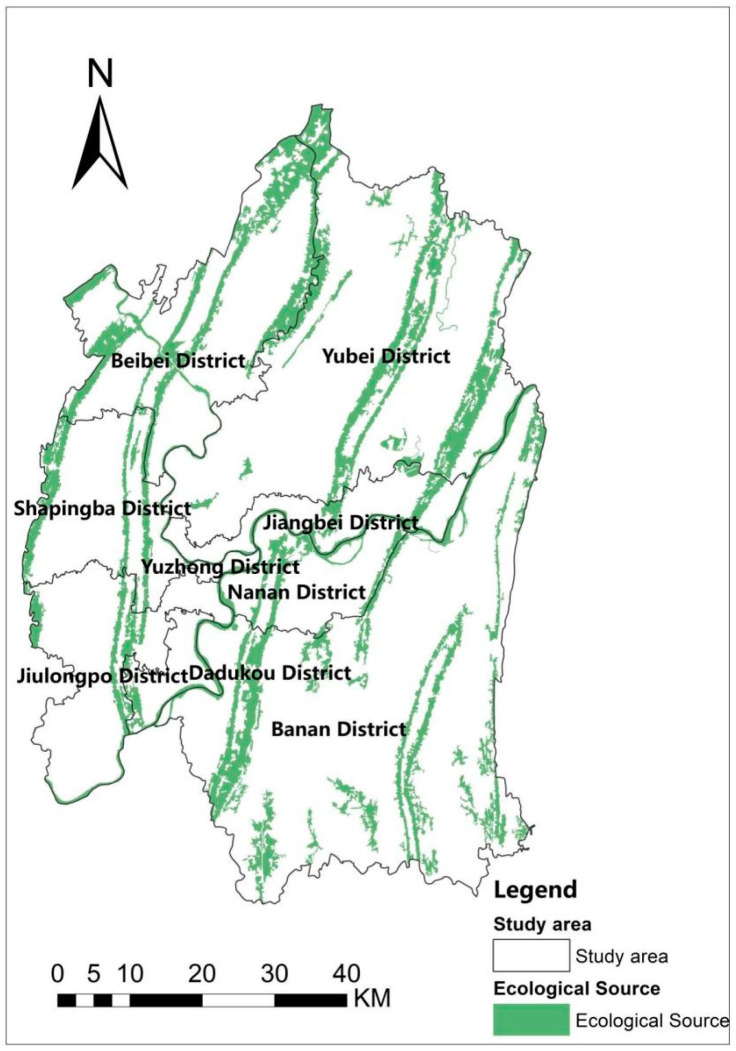
Ecological source.

The parameters of Formula (1) and Table 2 and Table 3 are combined with the InVEST model to obtain an ecological quality distribution map. Then, the natural breaks method is applied in which the distribution map can be effectively divided into classes I (extreme high), II (high), III (medium), IV (low) and V (very low) (Figure 4a). High ecological quality is positively correlated with high suitability, and collectively both contribute to the increased potential for ecological sources. Finally, the distribution map of the MSPA landscape pattern in the central city of Chongqing (Figure 4b) is derived from the analysis conducted by Guidos Toolbox. By combining the Class I areas with the highest ecological quality and the core areas of the MSPA, the team derives an area of 1240.15 km^2^. As patches below 2 km^2^ have a less ecological role, patches above 2 km^2^ are screened as ecological sources. Therefore, Figure 5 output from the above step is the distribution map of ecological sources, which consists of 43 land patches with 986.56 km^2^ and accounts for 79% of the combined core area. These ecological sources form a linear distribution with a multi-patch scattering pattern. The regular linear distribution comprises mountain ranges crossing the central urban area from north to south, intersecting with the Yangtze and Jialing rivers from east to west. Ecological sources are evenly dispersed in the central city of Chongqing, partly due to its mountainous distribution characteristics. In terms of subdivision, the metropolitan area has a more parallel distribution of mountain ranges at similar distances, which results in an approximately equidistant structure of ecological sources within the study area.

As shown in Table 7, the area of ecological sources is counted for each district. Yubei District and Banan District have the most extensive areas, with over 20% of the area in both. The area of ecological sources in Banan District is 291.46 km^2^, accounting for 29.54%; the area of ecological sources in Yubei District is 249.04 km^2^, accounting for 25.24%; followed by Beibei District, which has an area of 189.45 km^2^, accounting for 19.20%; the rest of the districts and counties have a comparatively low percentage, none of which exceeds 10%. Overall, the ecological sources of Chongqing’s central urban areas are mainly located in Banan, Yubei, and Beibei districts. The combined area of all three exceeds 75%, which plays a vital role in the regional ecosystem.

### 3.2. Ecological Corridor

**Figure 6:**
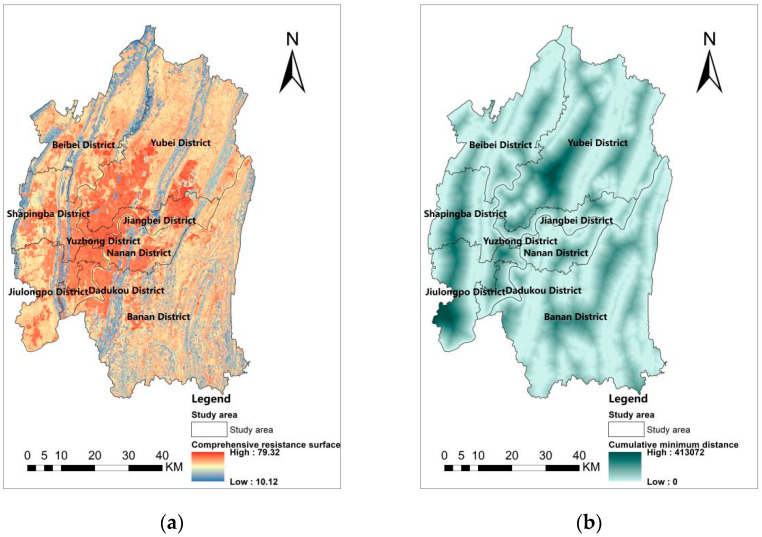
Comprehensive resistance surface and cumulative minimum distance (**a**) Comprehensive resistance surface (**b**) Cumulative minimum distance.

**Figure 7:**
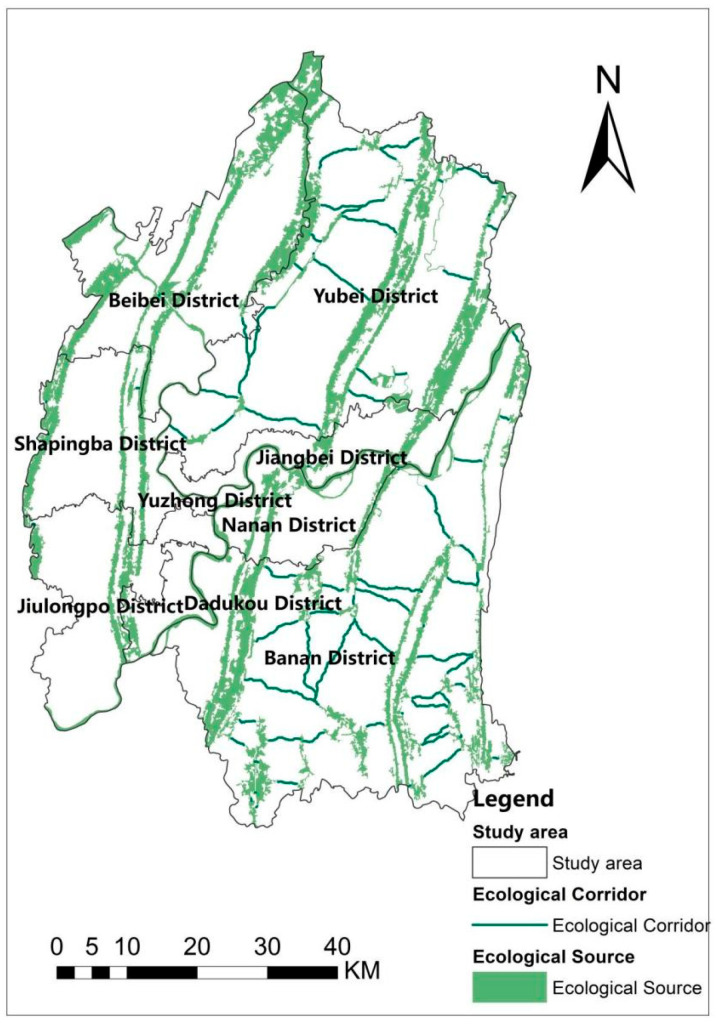
Ecological corridor distribution.

According to Table 5, the resistance factors are assigned and classified into categories using the ArcGIS 10.2 platform and combined with Equation (2) to obtain the cumulative minimum distance. The comprehensive resistance surface shows a high middle and low surrounding, it is influenced by complex topography, divided by mountains, with low resistance in the mountains and high resistance in the plains (Figure 6a). Figure 6b shows the range of cumulative minimum distance between 0 to 413,072. In addition, the map shows higher CWD in the southwestern part of Yubei District, the western part of Shapingba, the southwestern part of Jiulongpo, and the central part of Banan District. The corridor distribution map (Figure 7) shows 86 corridors within the test boundary, totaling 315.14 km, with a pattern of more in the east and less in the west, mainly in Yubei and Banan districts.

Statistics on the length of ecological corridors are shown in Table 8. They are mainly located in the Yubei and Banan districts of Chongqing, with the length of ecological corridors in the Banan district being 126.10 km, accounting for 40.01%; The length of ecological corridors in Yubei District is 182.66 km, accounting for 57.96%; the combined length of the two corridors represents more than 97% of the whole. The rest of the districts account for a minor proportion, with no more than 3%. This distribution characteristic is probably due to a large number of ecological source patches and fragmented distribution in the Yubei and Banan Districts.

### 3.3. Identification of Ecological Barrier Points and “Pinch Points“

#### 3.3.1. Identification of Ecological Barrier Points

**Figure 8:**
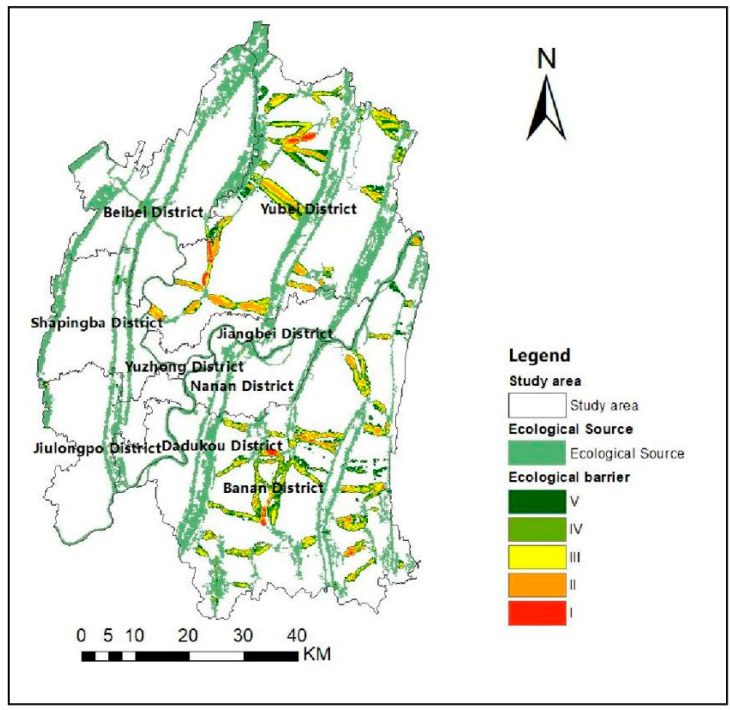
Classification of ecological barriers.

**Figure 9:**
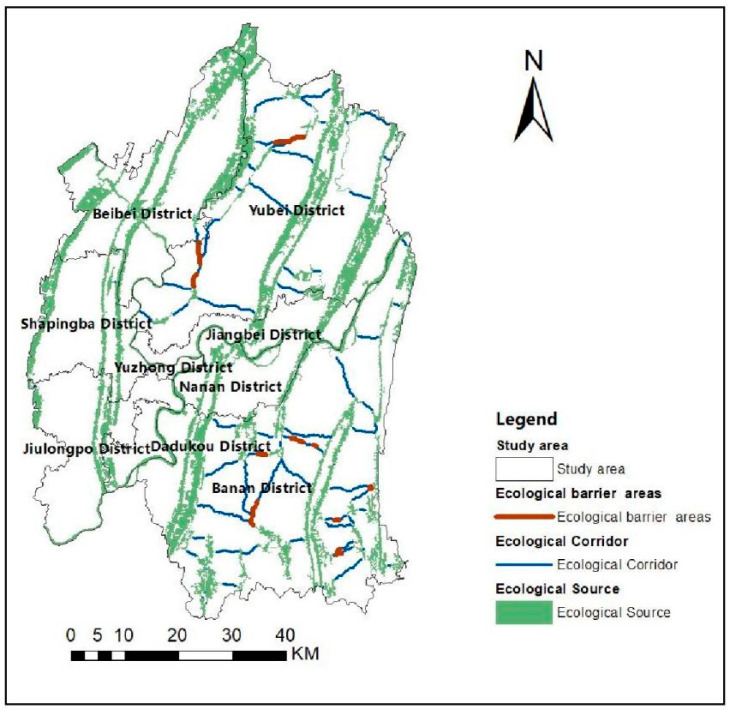
Distribution of ecological barrier points.

The circuit of ecological barrier point is analyzed using Barrier Mapper in Linkage Mapper Tools. First, the natural breaks method is used to classify the barriers into five classes Ⅰ (extreme high), Ⅱ (high), Ⅲ (medium), Ⅳ (low) and Ⅴ (very low). The higher the value of barrier points indicates more significant the obstruction to the ecological flow, where the area of classes I is the most considerable obstruction in the area. Figure 8 shows the classified map, indicating that the ecological barrier point areas of Class Ⅰ mainly exist in Jiangbei and Banan districts. Then, the ecological corridors are overlaid with the ecological barrier areas of class Ⅰ to obtain Figure 9. As shown in Figure 9, the two parts overlapping area is the barrier point of ecological corridors. Its removal can improve the effectiveness of the transfer at the ecological source, which is essential for the material transfer of the system.

Table 9 presents the statistics of the circuit areas of high barrier points on the ecological corridor. As one of the barrier areas straddles Beibei and Yubei districts, the statistics are segmented to facilitate the study. Therefore, the ecological barrier point areas illustrated in Table 9 are distributed in Beibei, Yubei, and Banan districts, with the most significant number in the Banan district. They consist of 13 sections with a length of 11.25 km, accounting for 46.49%, and these areas are numerous and scattered; Yubei District consists of 3 sections with a total length of 12.51 km, accounting for 51.69%, and they are more concentrated and less complex to restore than Banan District; Beibei District has an overall length of 0.44 km, accounting for 1.82%, and it is the least complicated to restore.

#### 3.3.2. Identification of Ecological “Pinch Points”

**Figure 10:**
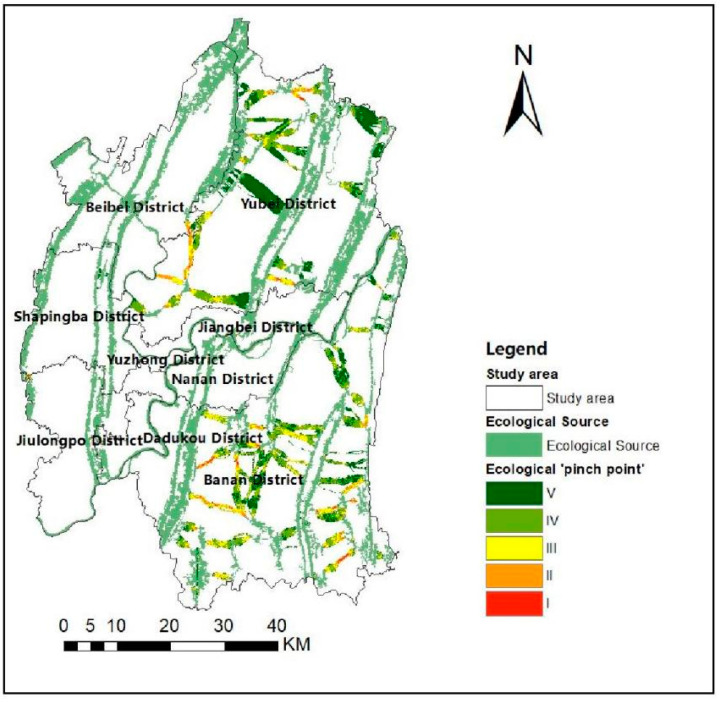
Ecological “pinch point” classification.

**Figure 11:**
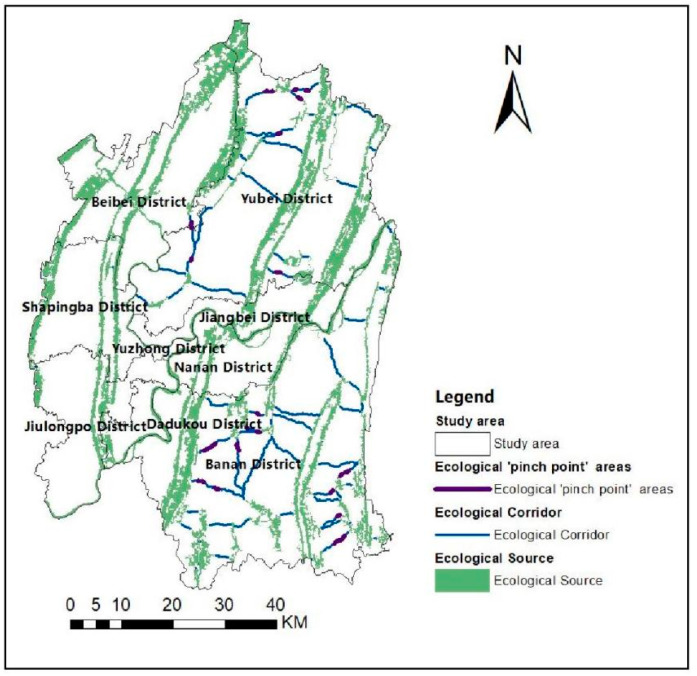
Ecological “pinch point” distribution.

The Pinch point Mapper in Linkage Mapper Tools is used to obtain a circuit map of the ecological “pinch points” distribution in the Chongqing metropolitan area and classify it into classes using the natural breaks method. Figure 10 shows it divided into five classes from high Ⅰ (extreme high) to low Ⅴ (extreme low). In addition, the circuit areas of Class I “pinch points” are superimposed on the ecological corridor to obtain a distribution map of the “pinch point” areas (Figure 11). As shown in Figure 11, the “pinch points” on the ecological corridor is mainly located in the north and southeast. These areas are vital to ecological stability, as damage to the ecological environment will affect the resilience of the whole system, and they need to be protected by discussing their actual conditions.

Table 10 shows the statistics of the areas where the ecological “pinch points” are located. According to the table, the three districts of Beibei, Yubei, and Banan are all spread out, with Banan being the primary area to be protected. It has the most significant number of 13 segments, with a total length of 12.66 km, representing 65.70% of the entire length. Yubei District is the following region as the secondary conservation zone with eight segments totaling 5.32 km in length, and this accounts for 27.61%. Finally, Beibei District, which is the least protected, has a length of 1.29 km, making up 6.69%.

## 4. Ecological Restoration Strategies

### 4.1. Barrier Removal

**Figure 12:**
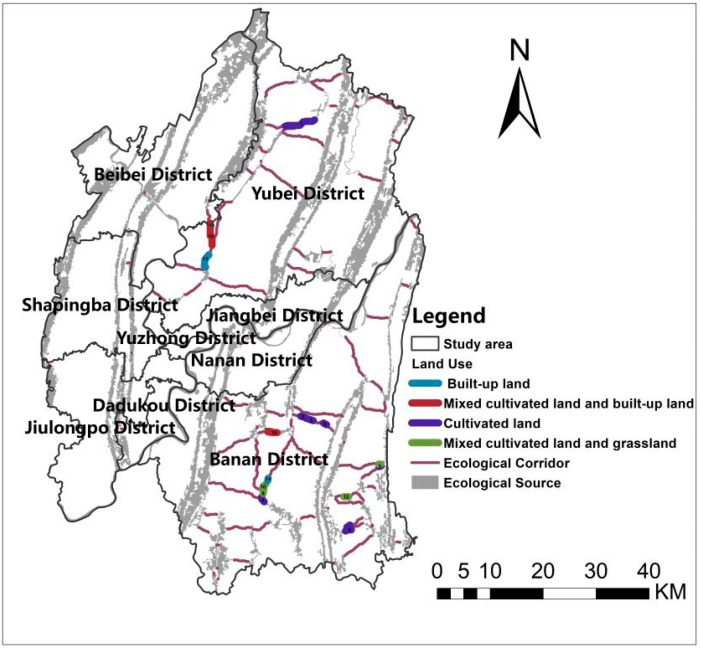
Types of land use at ecological barrier points.

The location of the ecological barrier point areas and the properties of the sites are visualized as shown in Figure 12. The land use types in the ecological barrier point areas could be divided into four main types: cultivated land, built-up land, mixed cultivated land and grassland, and mixed cultivated land and built-up land. Cultivated land accounts for the largest proportion of these four types, followed by built-up land, which reflects the landscape risk caused by the unbalanced spatial allocation after the significant increase in urbanization.

In Table 11, the ecological barrier points are numbered, and their land types and locations are counted. As land type is highly relevant to developing restoration strategies, it takes full consideration of the natural characteristics at the sites. The three mainland types are cultivated land, grassland, and built-up land. An appropriate return of the grain plots to forestry can be implemented according to local planting suitability for cultivated land and grassland. The selection of evergreen broad-leaved vegetation, such as yellow kudzu, balsam fir, and small-leaved ficus, which are highly adaptable to the environment, will gradually form a stable mixed evergreen broad-leaved forest. For barrier areas in built-up land, parkland could be created to increase the green space percentage in the built-up area, which also enhances the connectivity in the ecological corridor by constructing greenways of appropriate width. Therefore, areas containing both of these land types allow for various combinations of strategies to remove barrier sites.

### 4.2. Protection of “Pinch Points”

**Figure 13:**
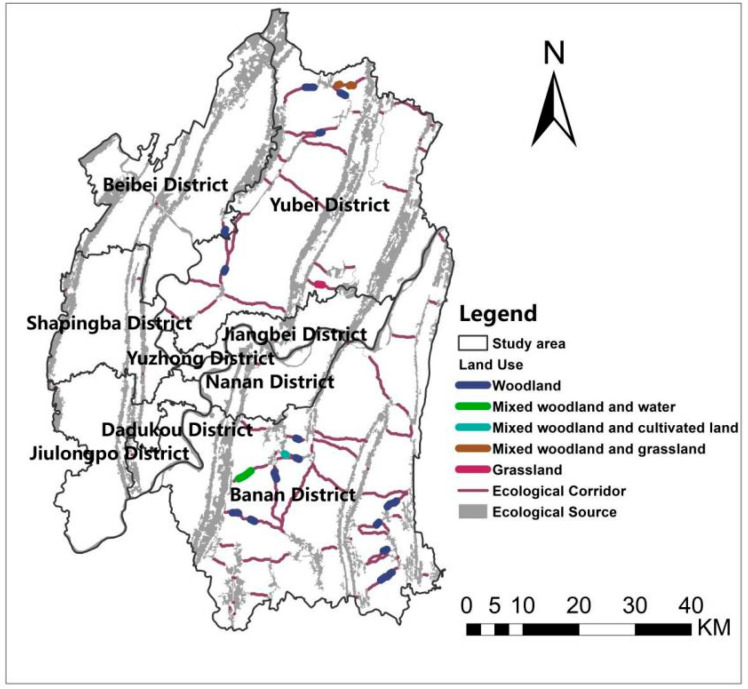
Types of land use at ecological “pinch points”.

As shown in Figure 13, the ecological “pinch points” areas are numbered, and their locations and types are visualized. The main types of land in the ecological ”pinch points” can be divided into five categories: grassland, woodland, mixed woodland and grassland, mixed woodland and cultivated land, and mixed woodland and water. In addition, woodland occupies the most significant proportion and is most likely to be mixed with other types, making it the most important to protect. The remaining types require additional protection of the corresponding land types in addition to the protection of woodland.

Table 12 describes where the ecological “pinch points” intersect with the corridor and the main land use types. After analyzing the ‘pinch point,’ made up of grassland, cultivated land, woodland, and water areas, conservation strategies will be established. For example, in the case of grassland and cultivated land, it is necessary to prohibit the harvesting of wild animals, reduce the use of pesticides and establish an environmental monitoring mechanism to monitor the ecological environment regularly; for woodland, a conservation mechanism should be developed to avoid over-exploitation of the woodland at the “pinch points” and to monitor the ecological condition on a regular basis. The restoration of water areas should focus on establishing a river protection system and reducing the disturbance of urban sewage. A combination of these strategies can be used to protect the “pinch points” in the face of multiple land types in the future.

## 5. Discussion

### 5.1. Comparison of Related Research

Urbanization and rapid population growth are exerting increasing pressure on urban ecosystems. Urban ecological security patterns are considered a framework for sustainable city development, and therefore the optimization and regulation of urban ecology based on ecological security patterns is a priority focus of the academic field nowadays. The regulation of the structure of ecological security patterns is the focus research. For example, Yang Junfu et al. proposed the ecological optimization framework of “two axes, four hearts, six belts and eight zones” based on the ecological security pattern of the Loess Plateau in China [18]; Wang Xinke et al. constructed the ecological security pattern of the eastern Minjiang River in China and proposed the ecological optimization framework of “two barriers, one belt, many corridors, and many spots”, and also emphasized They also emphasized the need to strengthen the connectivity of corridors and the protection of ecological sources [55]. Optimizing ecological security patterns through the landscape Index is also the research focus. For example, Ma Libang et al. constructed an ecological security pattern in the middle and lower reaches of the Shule River basin in China and selected 14 landscape pattern indices from six aspects: landscape area, landscape density, landscape shape, landscape proximity, landscape dispersion, and landscape diversity, and analyzed the relationship between them, and then proposed that the high degree of landscape fragmentation, landscape types and complex landscape patterns are conducive to ecological security [57]. In general, traditional studies are more inclined to macroecological regulation recommendations, which have important implications for ecological planning, but these optimization recommendations may be challenging to implement because they are too macro. This time, the key points’ type, number, and location can be determined through the ecological security pattern and circuit theory, which has operability characteristics and can be an essential supplement to ecological planning.

### 5.2. Characteristics of Ecological Security Patterns

The lack of topographic constraints in the cities of the plain has led to their continuous outward expansion so that their ecological sources are distributed in a pattern of few urban centers and high suburban areas, such as the ecological security pattern of Zhengzhou constructed by Dong Rencai et al. whose ecological sources are mainly distributed in suburban areas [58]; Ningbo city is the same example, and Yu Han et al. constructed an ecological security pattern of Ningbo, China, with its ecological source sites showing a distribution pattern of high in the middle and low around [59]. First, the topography of the mountainous city is more complex than the cities of the plains, which results in it retaining a large amount of mountainous land that is unsuitable for construction. These mountainous areas, which are less interfered with by humans, are critical ecological sources of the study area. Secondly, water systems are also essential components of ecological sources. From a macroscopic perspective, the sources of mountain cities comprise two significant components: mountains and water systems. Therefore, the core of protecting the ecological sources of mountain cities is to protect the integrity of mountains and water systems. The distribution of ecological source sites directly affects the distribution of corridors [60]. In the study, ecological corridors are mainly distributed in Banan and Yubei districts, and this distributed characteristic may be due to the many patches in the Yubei and Banan districts and their fragmented distribution. This allows for subsequent ecological optimization by modestly increasing the number of ecological source patches to adjust the ecological corridors.

### 5.3. Characteristics of Ecological Key Points

After analyzing the ecological barrier points, the most common type of land use is cultivated land, followed by built-up land, indicating that the main factors causing barriers to ecological migration in Chongqing are the expansion of cultivated land and urban sprawl. Current studies have less discussion on the land use of ecological barrier sites, but a large number of studies have shown that the expansion of cultivated and built-up land is the most critical factor contributing to ecological degradation, which is consistent with the results of the existing study [61,62]. Cultivated land has specific ecological attributes and only requires reasonable local population management. In contrast, the barrier points are more challenging to remove for built-up land and require more costly adjustments by adding green areas or changing corridor paths. The “pinch points” are mainly made up of woodland, indicating that woodland acts as ecological stepping-stones in the study area and that the protection of these areas is vital for the overall ecological pattern.

### 5.4. Deficiencies and Further Research Directions

Based on the principles of landscape ecology, this paper identifies ecological sources and corridors in the central city of Chongqing, a typical mountainous city, and then evaluates the ecological barriers and “pinch points” in the ecological corridors through circuit theory and proposes protection and restoration strategies by summarizing the types of key points. While the study can accurately identify the location, type, and scale of key points on the ecological corridor, there are limitations to this exploration since land use is not the only factor affecting ecology for optimizing land use at key points. Conditions such as building scale, tree species, and the land mix should also be addressed in further research, and their interaction should be considered.

## 6. Conclusions

The construction of ecological security patterns has an important impact on clarifying the ecological status within the study area [63,64]. In this study, the ecological security pattern of the study area is studied through the application of the MSPA model, the INVEST model, and MCR analysis. As a result, it is found that there are 46 ecological sources in the central city of Chongqing, which are comparatively distributed and consist mainly of mountains and water systems that are important for maintaining the sustainability of the study area. In the center of Chongqing, there are 86 ecological corridors, with a pattern of more east than west, which is significant for the migration of animals and should be avoided in future construction.

Protecting and restoring key points can improve ecological quality at a relatively lower budget [65,66]. Based on the study of ecological patterns, circuit theory is applied to identify the key points in the ecological corridors. The research found 17 sites totaling 24.20 km of the ecological corridor in the barrier point zone. It consists mainly of build-up land and cultivated land. In addition, up to 22 segments, totaling 19.27 km of the ecological corridor, are located in the pinch point zone. It is composed mainly of woodland. The project provides statistics on these key points’ location, type, and scale, which will facilitate the reallocation of resources in subsequent practical works.

In general, the ecological security pattern of mountainous cities is mainly composed of mountains and water systems, so the protection of mountains and water systems is vital in mountainous cities, and the integrity of mountains and water systems should be ensured as much as possible in the process of urban development. At the same time, the expansion of construction land and cultivated land in mountainous cities may be the most crucial factor causing ecological barrier points, and ecological “pinch points” are primarily composed of woodland. Therefore, ecological restoration can be carried out by means of afforestation.

## Figures and Tables

**Table 1:** Data source.

Data Type	Date	Precision	Data Source
Land Use	2020	30 × 30 m	National Basic Geographic Information Center Global Surface Coverage Data Product Service Website (http://www.globallandcover.com/ accessed on 1 June 2022)
ASTER GDEM v3	2019	30 × 30 m	Geospatial Data Cloud (http://www.gscloud.cn/ accessed on 3 June 2022)
Landsat 8 OLI_TIRS	2021	30 × 30 m	Geospatial Data Cloud (http://www.gscloud.cn/ accessed on 5 June 2022)
Road Network	2022	Shp	Open Street Map (https://www.openstreetmap.org/ accessed on 15 June 2022)
Administrative Boundaries	2019	Shp	Chongqing Territorial Spatial Planning (2019–2035)

**Table 2:** Maximum influence distance and weight of the threat factors.

Threats	Maximum Influence Distance/km	Weight	Spatial Decay Type
Urban land	10	1	Exponential
Residential Locations	6	0.6	Exponential
Railway	4	0.5	linear
Highway	3	0.4	linear
Cropland	8	0.7	linear

**Table 3:** Sensitivity of different habitat types to different threat factor.

Land-Use	Habitat	Threat Factors				
		Urban Land	Residential Locations	Railway	Highway	Cropland
Cropland	0.3	0.6	0.4	0.5	1	0
Woodland	1	0.9	0.8	0.8	0.7	0.8
Grassland	0.9	0.6	0.5	0.5	0.6	0.5
Shrub Wood	0.9	0.7	0.6	0.6	0.5	0.6
Wetland	0.8	0.8	0.6	0.5	0.4	0.7
Water	1	0.7	0.6	0.5	0.4	0.1
Urban land	0	0	0	0	0	0
Bare land	0.3	0.1	0.1	0.2	0.2	0.1

**Table 4:** Landscape types of MSPA and their meanings.

Landscape Type	Ecological Meaning
Core	Large habitat patches that can serve as source areas and provide habitats or migration places for wildlife
Islet	Small patches that are weakly connected to each other, providing a place for species to spread and communicate and promoting the flow of matter and energy
Perforation	Transition zone between the core area and the non-green landscape area: the edge of the internal patch, which has edge effects
Edge	Transition zone between the core area and the non-green landscape area; has an edge effect and protects the ecological process of the core area
Bridge	Connecting corridor of the adjacent core area; provides the necessary pathways for species diffusion and energy exchange between adjacent patches of core areas
Loop	Connects corridors inside the same core area to provide access to species diffusion and energy exchange within the core patch
Branch	Only one side is connected to an edge, bridge, loop or perforation

**Table 5:** Resistance factors and classification.

Resistance Factor	Very Low	Low	Medium	High	Extreme High	Weights
	10	30	50	70	90	
Land use	Woodland	Grassland	Water	Cultivated land	Construction Land	0.41
Implicit ecological resistance values			kriging			0.13
Slope	8	8–15	15–25	25–35	35	0.17
Topographic relief	0–25	25–50	50–75	75–100	100	0.15
NDVI	0.8–1	0.6–0.8	0.4–0.6	0.2–0.4	0–0.2	0.14

**Table 6:** Linkage Mapper Tools choice.

Tools	Applications
Barrier Mapper	Implements a new method for detecting important barriers to facilitate restoration planning.
Pinchpoint Mapper	Uses Circuitscape to identify pinch-points in corridors produced by Linkage Mapper.

**Table 7:** Ecological source area statistics.

Name	Area (km^2^)	Percentage (%)
Yubei District	249.04	25.24
Shapingba District	75.88	7.69
Nanan District	55.38	5.61
Jiulongpo District	53.30	5.40
Jiangbei District	46.03	4.67
Dadukou District	21.03	2.13
Beibei District	189.45	19.20
Banan District	291.46	29.54
Yuzhong District	4.99	0.51
Total	986.56	100

**Table 8:** Ecological corridor length statistics.

Name	Length (km)	Percentage (%)
Yubei District	126.10	40.01
Shapingba District	0.61	0.20
Nanan District	0.22	0.07
Jiulongpo District	0.70	0.22
Jiangbei District	0.09	0.03
Dadukou District	0.18	0.06
Beibei District	4.58	1.45
Banan District	182.66	57.96
Yuzhong District	0.00	0.00
Total	315.14	100.00

**Table 9:** Length statistics of ecological barrier points.

Name	Quantity	Length (km)	Percentage (%)
Beibei District	1	0.44	1.82
Yubei District	3	12.51	51.69
Banan District	13	11.25	46.49
Total	17	24.20	100.00

**Table 10:** Ecological “pinch point” length statistics.

Name	Quantity	Length (km)	Percentage (%)
Beibei District	1	1.29	6.69
Yubei District	8	5.32	27.61
Banan District	13	12.66	65.70
Total	22	19.27	100.00

**Table 11:** The length and location of ecological barrier points.

No.	Length (km)	Land Use	Location
1	5.87	Cultivated land	Southwest of Dawan Town, Yubei District
2	1.11	Cultivated land	Northeast of Huimin Street, Banan District
3	0.87	Cultivated land	East of Huimin Street, Banan District
4	1.12	Cultivated land	Southwest of Nanpeng Street, Banan District
5	0.41	Cultivated land	Southeast of Dongquan Town, Banan District
6	1.14	Cultivated land	East of Jilong Town, Banan District
7	0.67	Cultivated land	East of Jilong Town, Banan District
8	0.64	Cultivated land	West of Jiangjia Town, Banan District
9	0.48	Mixed cultivated land and grassland	Southwest of Nanpeng Street, Banan District
10	1.30	Mixed cultivated land and grassland	Southwest of Nanpeng Street, Banan District
11	0.39	Mixed cultivated land and grassland	Southeast of Dongquan Town, Banan District
12	0.90	Mixed cultivated land and grassland	East of Jilong Town, Banan District
13	2.73	Built-up land	Central Cuiyun Street, Yubei District
14	0.45	Built-up land	South of Nanpeng Street, Banan District
15	4.35	Mixed cultivated land and built-up land	South of Fuxing Town, Beibei District
16	1.77	Mixed cultivated land and built-up land	North of Nanpeng Street, Banan District

**Table 12:** The length and location of ecological “pinch points”.

No.	Length (km)	Land Use	Location
1	0.90	Grassland	Southwest of Longxing Town, Yubei District
2	1.46	Woodland	Northwest of Nanpeng Street, Banan District
3	0.65	Woodland	Northeast of Nanpeng Street, Banan District
4	0.60	Woodland	Central Huimin Street, Banan District
5	2.55	Woodland	Northwest of Shitan Town, Banan District
6	0.69	Woodland	East of Jilong Town, Banan District
7	0.66	Woodland	West of Shilong Town, Banan District
8	0.65	Woodland	Northwest of Shilong Town, Banan District
9	0.81	Woodland	Northwest of Shilong Town, Banan District
10	0.79	Woodland	Northwest of Jumping Stone Town, Banan District
11	0.40	Woodland	South of Jieshi Town, Banan District
12	0.34	Woodland	South of Jieshi Town, Banan District
13	0.63	Woodland	Northeast of Cuiyun Street, Yubei District
14	1.29	Woodland	South of Fuxing Town, Beibei District
15	0.61	Woodland	East of Tizhu Town, Yubei District
16	1.03	Woodland	Northwest of Dawan Town, Yubei District
17	0.57	Woodland	East of Xinglong Town, Yubei District
18	0.40	Woodland	Northeast of Zizhu Town, Yubei District
19	0.73	Mixed woodland and grassland	North of Dawan Town, Yubei District
20	0.45	Mixed woodland and grassland	Northwest of Zizhu Town, Yubei District
21	0.39	Mixed woodland and cultivated land	Northeast of Nanpeng Street, Banan District
22	2.67	Mixed woodland and water	Middle of Jieshi Town, Banan District

## Data Availability

The data presented in this study are available on request from the corresponding author. The data are not publicly available due to privacy.
